# Immunity to Protozoan Parasites

**DOI:** 10.1155/2012/250793

**Published:** 2012-05-03

**Authors:** Marcela F. Lopes, Dario S. Zamboni, Hugo D. Lujan, Mauricio M. Rodrigues

**Affiliations:** ^1^Instituto de Biofísica Carlos Chagas Filho, Universidade Federal do Rio de Janeiro, 21941-902 Rio de Janeiro, RJ, Brazil; ^2^Departamento de Biologia Celular Molecular e Bioagentes Patogenicos, Faculdade de Medicina de Ribeirão Preto, Universidade de São Paulo, 14049-900 Ribeirão Preto, SP, Brazil; ^3^Laboratorio de Bioquímica y Biología Molecular, Facultad de Medicina, Universidad Católica de Córdoba, X5004ASK Cordoba, Argentina; ^4^CINTERGEN, Escola Paulista de Medicina, UNIFESP, 04044-010 São Paulo, SP, Brazil

Protozoan parasites cause several diseases, such as Malaria, Leishmaniasis, and Trypanosomiasis, hampering human development worldwide. Many protozoa cause infections that often follow chronic courses, owing to coevolution between parasites and host immune system. The survival and transmission of pathogenic protozoa depends on their ability to evade or subvert host's innate and adaptive immune responses. A great challenge to research in immunology and parasitology is the development of strategies that favor immunity against protozoan parasites and prevent their evasion, chronic, or recurrent infections and associated pathologies. This special issue includes original papers and reviews that summarize current advances in our understanding on the mechanisms of immunity to protozoan parasites in humans and experimental animal models.

The discovery of pattern recognition receptors (PRRs) devoted to detection of pathogen- or microbe-associated molecular patterns (PAMPs/MAMPs) fostered a vibrant research in the past decade, identifying new receptors, new ligands on pathogens, and key signaling pathways inducing innate immunity [1–3]. Therefore, it is not surprising that about 70% of the papers published here deal with early steps of immune responses to protozoan infections, including the role of parasite products, host receptors, molecular mechanisms, and effector cells of innate immune system.

Four papers of this special issue discuss molecules present at initial encounter between hosts and protozoan parasites plus the secretions of their insect vectors. Macrophage migration inhibitory factor (MIF, “*Macrophage migration inhibitory factor in protozoan infections*”) is a host inflammatory cytokine with protective or pathogenic actions in distinct protozoan infections. Interestingly, parasites also express their own MIFs, a relevant finding that deserves further investigation. During transmission of *Trypanosoma cruzi*, lysophosphatidylcholine (LPC, “*Lysophosphatidylcholine: a novel modulator of Trypanosoma cruzi transmission*”) is introduced with vector's secretions and recruits host cells, promoting infection. Apoptotic cells also express LPC among other “find me” and “eat me” signals that favor their clearance by professional phagocytes. As described elsewhere, interactions between apoptotic cells and *T. cruzi*-infected macrophages induce intracellular parasite replication [4]. Similarly, *Leishmania amazonensis* parasites express phosphatidylserine (PS, “*Subversion of immunity by Leishmania amazonensis parasites: possible role of phosphatidylserine as a main regulator*”), mimicking apoptotic cells to inhibit host cell activation and exacerbate infection. In “*New insights on the inflammatory role of Lutzomyia longipalpis saliva in Leishmaniasis*”, the authors discuss how immunization against vector's saliva components might help development of a new approach to prevent Leishmaniasis. Further research will be required to define target antigens involved in distinct biological activities of vector's saliva for novel vaccines against *Leishmania spp*. infections.

Another four papers review the role of Toll-like receptors (TLRs) on immunity to parasitic infections, their ligands, and how this knowledge can be translated into PAMP-based adjuvants for novel vaccine strategies. Authors present what we have learnt from experiments performed in mice defective in one or multiple TLRs or key signaling molecules to gain insight into their role on immunity triggered by protozoan PAMPs, with emphasis on *T. cruzi* and *Leishmania* infections. Other PRRs, such as Nod-like receptors (NLR), only recently came into spotlight in immunity to protozoan parasites [5]. Lack of putative PRRs not only affects innate immunity, but also the development of certain adaptive immune responses, presumably due to their role on antigen-presenting dendritic cells (DCs). This is particularly relevant for new vaccine approaches aiming at inducing memory T cells against intracellular protozoan infections (*The immune response to Trypanosoma cruzi: role of toll-like receptors and perspectives for vaccine development*). Whereas protozoan PAMPs activate innate immunity through TLR-induced signaling pathways, other molecules, such as lipophosphoglycan (LPG, “*Innate immune activation and subversion of mammalian functions by Leishmania lipophosphoglycan*”), subvert the immune responses, allowing parasite escape (*Toll-like receptors in Leishmania infections: guardians or promoters*). Furthermore, detection of protozoan PAMPs by immune and target tissue cells can induce protective, regulatory, or even pathogenic reactions upon infection (*Nonimmune cells contribute to crosstalk between immune cells and inflammatory mediators in the innate response to Trypanosoma cruzi infection*). How responses to stress occur in pathogenic protozoa and host tissues has been a topic of increasing interest for development of drugs to treat parasitic diseases [6].

There are also three papers that address the major effector cells in innate immunity to protozoan parasites: neutrophils, macrophages, dendritic cells, and their arsenal of effector molecules. Lately, monocytes were also imputed as major effector and regulatory players against protozoan parasites [7, 8]. ETosis was recently described in neutrophils as a death process leading to extrusion of antimicrobial DNA traps, which can also be triggered by and act on protozoan parasites (*ETosis: a microbicidal mechanism beyond cell death*). In “*Reactive oxygen species and nitric oxide in cutaneous Leishmaniasis*”, the authors describe effector molecules expressed by phagocytes and subsets of macrophages, with protective or regulatory roles in protozoan infections. In “*The role of vitamin D and vitamin D receptor in immunity to Leishmania major infection*”, the authors take advantage of deficiency in vitamin D receptor to show that vitamin D controls susceptibility to *Leishmania major* infection in resistant, but not susceptible mice. Vitamin D receptor signaling seems to inhibit the ability of DCs both as effector cells and as promoters of Th1-protective adaptive immunity.

In four papers of this issue, the authors approach the role of adaptive immunity in both protection and pathogenesis of protozoan infections. “*Thymus atrophy and double positive escape are common features in infectious diseases*” and “*B cell response during protozoan parasite infections*” discuss how protozoan parasites negatively affect development and modulate functions of T and B lymphocytes in cellular and humoral responses to infections. In “*Evidence for T cell help in the IgG response against tandemly repetitive Trypanosoma cruzi B13 protein in chronic Chagas disease patients*”, authors investigated how the cooperation between T and B cells result in humoral responses to a parasite antigen in chronic chagasic patients. In “*Comparison of protective immune responses to apicomplexan parasites*”, the researchers focus on the immune responses to apicomplexan protozoa, including *Plasmodium*, *Toxoplasma*, and *Cryptosporidium*. They also discuss issues such as antigenic variation that precludes development of conventional or transmission vaccines against these infections. Recently, disruption of the mechanism of antigenic variation in the protozoan *Giardia lamblia*, which is regulated posttranscriptionally by an RNA interference-like mechanism [9], allowed the formulation of an effective vaccine against this intestinal parasite [10].

In another paper, the researchers propose a mathematical model to predict Malaria transmission in endemic areas under drug pressure, taking into account induction of relapses by *Plasmodium vivax* infection, which confer the ability to boost adaptive immunity and prevent clinical Malaria.

Hopefully, the strong body of information available in this special issue will facilitate research on new mechanisms of immunity as well as the development of novel vaccine and immunotherapeutic tools against parasitic diseases, which are some of the most deadly human infections. 

*Marcela F. Lopes* *Marcela F. Lopes* *Dario S. Zamboni* *Dario S. Zamboni* *Hugo D. Lujan* *Hugo D. Lujan* *Mauricio M. Rodrigues* *Mauricio M. Rodrigues*
